# Can endobronchial or endotracheal metastases appear from rectal adenocarcinoma?

**Published:** 2017

**Authors:** GL Serbanescu, RM Anghel

**Affiliations:** * Prof. Dr. Al. Trestioreanu” Institute of Oncology, Bucharest, Romania;; ** “Carol Davila’’ University of Medicine and Pharmacy, Bucharest, Romania

**Keywords:** rectal cancer, endobronchial/ endotracheal metastasis, radiotherapy

## Abstract

**Background:** Endobronchial and endotracheal metastases from extra-pulmonary solid tumors are rare.

**Patients and methods:** We reported the case of a patient diagnosed with endobronchial and endotracheal metastases from rectal adenocarcinoma.

**Case report:** Patient P.G., 62 years old, was diagnosed with a rectal tumor in 2011, for which, a surgical intervention was performed (pT3 pN2a M0, stage IIIB). Afterwards, she underwent adjuvant chemotherapy and concomitant radiochemotherapy.

In September 2013, the chest CT showed 2 nodules for which, an incomplete surgical resection was done and which were histopathologically diagnosed as metastases from rectal cancer. The patient continued the treatment with chemotherapy associated with Bevacizumab and after 6 months only Bevacizumab for maintenance.

In June 2015, the chest CT pointed out a nodule in the right upper lobe and the bronchoscopy highlighted a 4-5 mm lesion at the level of the right primary bronchus, whose biopsy proved the rectal origin. Afterwards, another surgical intervention was performed. Unfortunately, the postoperative chest CT revealed an intratracheal tissue mass (11/ 7mm) and multiple metastases in the right lung. The bronchoscopy showed 2 endotracheal lesions, out of which one was biopsied (histopathological result of metastasis from rectal cancer). Despite the fact that chemotherapy was continued, other endobronchial lesions appeared. All of them were removed and the patient started radiotherapy on the tracheal area. Afterwards, she refused to continue chemotherapy. The last bronchoscopy highlighted one endobronchial and two endotracheal secondary malignant lesions.

**Conclusion:** Endobronchial and endotracheal metastases must be taken into consideration in all the patients with a history of extra-pulmonary cancer.

**Abbreviations:** CT = computed tomography, MRI = magnetic resonance imaging, IMRT = intensity-modulated radiotherapy, ESMO = European Society for Medical Oncology, NCCN = National Comprehensive Cancer Network, iv = intravenous, PET – CT = Positron Emission Tomography – Computed Tomography

## Introduction

Endobronchial and endotracheal metastasis secondary to extra-pulmonary solid tumors or to primary lung tumors are rare, but can be life-threatening [**1**]. 

Among the different localizations of the tracheobronchial tree, the trachea represents an extremely rare site for metastasis from solid extra-pulmonary cancers, being involved in only 5% of the cases [**1**]. The most frequent solid primary non pulmonary tumors which determine the appearance of endobronchial/ endotracheal metastasis are breast cancer, colorectal cancer, renal cell carcinoma and malignant melanoma [**1**,**2**].

## Patients and methods

The intrabronchial and intratracheal involvement were histopathologically proven as sites for metastasis from rectal cancer.

## Case report

The case of a 62-years-old Caucasian female patient, P.G., non-smoker, who denied alcohol beverages consumption, did not work in a toxic environment and who lived in the urban area, was presented. Personal physiologic antecedents were menarche at 12 years, one natural childbirth, and menopause at 50 years old. Family and personal pathologic history were without importance for the malignancy. The debut of the neoplasia was in 2011 with the rectal bleeding and lower abdominal pain for 2 to 3 months before she presented to the doctor. Colonoscopy showed an ulcerated non-obstructive tumor above 10 cm from the anal verge which was biopsied and whose histopathological result was uncertain (adenocarcinoma/ high-grade intraepithelial neoplasia). The patient underwent an abdominal and pelvic MRI with intravenous contrast which confirmed the presence of a rectal tumor (3/ 4,2 cm), which infiltrated the locoregional fat tissue and also showed infracentimetric locoregional lymph nodes. The chest CT showed no pathological changes. The CEA biological marker, the complete blood count, and the chemistry profile were within normal limits.

In September 2011, the surgical intervention was performed and consisted in a low anterior rectal resection. The histopathological result was of a well-differentiated adenocarcinoma with 20 resected lymph nodes from which 4 presented tumoral invasion (pT3pN2a, Ro).

So, in October 2011, the patient presented to “Prof. Dr. Al. Trestioreanu” Institute of Oncology in Bucharest with a good performance status (ECOG = 0) and the diagnosis of operated rectal adenocarcinoma stage IIIB. According to the NCCN and ESMO guidelines, she received adjuvant chemotherapy type FOLFOX6 (Oxaliplatin 85mg/ m2 iv day 1 + leucovorin 400mg/ m2 iv day 1 + 5-FU 400mg/ m2 iv bolus on day 1 and 5-FU 2400mg/ m2 over 46 hours iv continuous infusion, at every 2 weeks) for 6 months, which was well tolerated and in full dose. The imagistic tests and the level of CEA after 3 and 6 months of adjuvant chemotherapy (abdominal and pelvic CT with intravenous contrast) were within normal limits.

Afterwards, according to the NCCN and ESMO guidelines, between June and July 2012, the patient underwent external beam radiotherapy up to a total dose of 50,4 Gy on the tumoral bed and locoregional pelvic lymph nodes (daily 1,8 Gy fractions, 5 days per week), concomitant with chemotherapy based on Capecitabine 825mg/ m2 twice a day, at 30 min after main meals, for 5 days per week. As side effects, we noticed diarrhea, grade 1 leucopenia and neutropenia in the last week of treatment.

Until September 2013, the follow-up consisted of a complete physical examination and CEA level determination at every 3 months, chest/ abdominal/ pelvic CT with intravenous contrast at every 6 months and colonoscopy every year. Then, the patient was asymptomatic, but the chest CT revealed 2 lung nodules localized in the right upper and right lower lobe, with dimensions of 12/ 11/ 11 mm, 6/ 7/ 7 mm respectively, for which, an incomplete resection was performed. The histopathological result was lung metastasis of adenocarcinoma with intestinal origin, without information about the resection margins. Therefore, immunochemistry interpretation was done and confirmed the result of a well-differentiated lung metastasis secondary to rectal cancer. The molecular analysis for mutation detection in exons 2, 3, 4 of the K-RAS and N-RAS oncogenes showed that the tumor carried a mutation in exon 2 codon 12 of K-RAS oncogene. The CEA level, the complete blood count, and the chemistry profile were within normal limits.

The oncological treatment was continued with chemotherapy type FOLFIRI (Irinotecan 180mg/ m2 iv day 1 + leucovorin 400mg/ m2 iv day 1 + 5-FU 400mg/ m2 iv bolus on day 1 and 5-FU 2400mg/ m2 over 46 hours iv continuous infusion, at every 2 weeks) in association with Bevacizumab 5mg/ kgc iv, day 1, at every 2 weeks for 6 months and afterwards only Bevacizumab for maintenance, very well tolerated.

The chest CT from June 2015 revealed a lung nodule localized in the right upper lobe with a suggestive aspect for metastasis. The bronchoscopy highlighted a lesion of 4-5 mm on the right primary bronchus, with a good vascularization, surrounded by normal mucosa and from which biopsy was taken. The histopathological result was metastasis of rectal adenocarcinoma. The sample was too small for immunochemistry tests. Further, the patient was investigated with a PET-CT which showed a metabolic activity for a lung nodule from the right upper lobe with a dimension of 2,9 cm and a max of 13,13 SUV; it did not offer any information about the tracheobronchial tree.

The patient was proposed for another surgical intervention, but she refused it. She returned in November 2015 with a CT scan and a bronchoscopy similar to those in June 2015. In December 2015, an upper right lobectomy with a resection of the primary bronchus and part of the intermediate bronchus was performed. The histopathological result confirmed that the resected pieces contained lesions of multifocal colon-like adenocarcinoma.

**Fig. 1:**
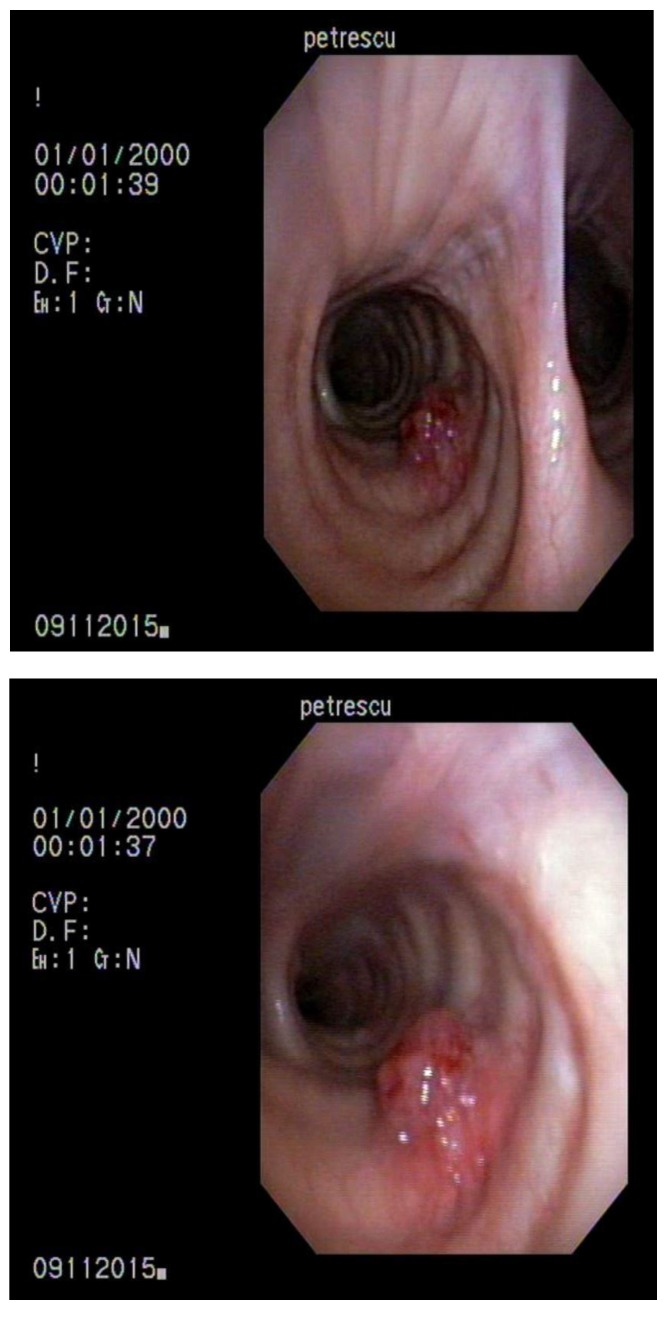
November 2015 – Lesion localized in the right primary bronchus with suggestive aspect for metastasis

After this complex surgical intervention, the patient presented a poor performance status and until February 2016 interrupted any oncological treatments. Then, the chest CT pointed out multiple secondary nodules in the right intermediate and lower lobes and an intratracheal mass with the dimension of 11/ 7mm. The CEA level was within normal values. Unfortunately, the patient presented intermittent nonproductive cough and stridor. The bronchoscopy highlighted 2 endotracheal lesions: one of 1,5 mm and the other one of 1 cm, which was biopsied and proved to be metastasis from rectal cancer. The patient continued chemotherapy with Oxaliplatin (85mg/ m2 iv) and Irinotecan (200mg/ m2) at every 3 weeks. In March 2016, through total anesthesia, one tracheal lesion was resected and for other three, local laser therapy was performed. In April 2016, the patient performed chest/ abdominal/ pelvic CT with intravenous contrast, which showed stable lung nodules localized in the right intermediate and lower lobes and no pathological information about the tracheobronchial tree. Therefore, she continued chemotherapy with Irinotecan and Oxaliplatin. As side effects, we remarked peripheral sensory neuropathy and grade 1-2 leucopenia and neutropenia. The bronchoscopy in May 2016 showed a right bronchial tree without alterations on the mucosa, but on the left one, 2 lesions of 3 mm and 2 mm, which were resected. 

Between June and July 2016, the patient underwent external beam radiotherapy, using the IMRT technique, up to a total dose of 50,4 Gy to the tumoral bed with a conventional fractionation schedule. The treatment was well tolerated, with moderate dysphagia and the relief of the respiratory symptoms appeared.

Since the end of radiotherapy, the patient refused to continue chemotherapy. 

However, at the end of October, the bronchoscopy showed 2 endotracheal lesions (of 5 and 8 mm) and one on the left primary bronchus with an aspect suspected for metastases, which were resected and their histopathological result proved the rectal origin.

**Fig. 2:**
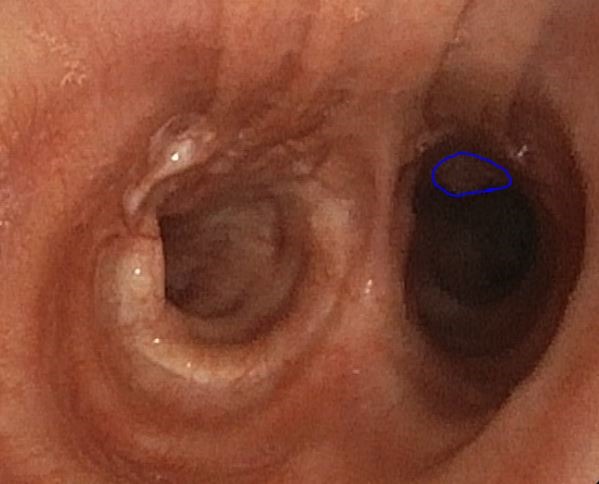
October 2016 - Intrabronchial lesion

## Discussion

The particularity of this case was represented, firstly, by the fact that endobronchial and endotracheal metastases, which are a rare condition [**1**], have occurred in the evolution of a rectal adenocarcinoma. Usually, they appear late after the diagnosis of a primary tumor, at a median of 50 months, being associated with other metastases [**1**,**3**]. Our patient presented tracheobronchial tree metastases after 43 months from the diagnosis of a rectal cancer.

In this association with rectal cancer it is possible that they are underdiagnosed because bronchoscopy is the most important test through which the physician establishes the diagnosis of certitude, but it is not seldom used between the investigations that are done in a rectal cancer [**4**,**5**]. Through histopathology, it is very important to make the differential diagnosis with a primary lung cancer, because the treatment and prognosis differ [**5**-**7**]. As in the presented case, the symptoms that can appear in this condition can be life-threatening and are characteristic for an upper airway obstruction: cough, hemoptysis, dyspnea and stridor [**3**,**4**].

Regarding the treatment of endobronchial and endotracheal metastasis, usually it is palliative [**3**,**5**,**8**]. It can relief the symptoms which are due to the upper airway obstruction and improve the quality of life [**3**,**4**,**6**]. It is represented by surgery, chemotherapy, external beam radiotherapy, cryotherapy, and brachytherapy [**4**]. 

In the presented case, through surgery and chemotherapy the tracheobronchial metastasis continued to reappear very soon after their end. Radiotherapy succeeded in ameliorating the respiratory symptomatology and the interval between its end and the appearance of new lesions lasted longer.

Neoadjuvant treatment (radiotherapy +/ - chemotherapy) represents the standard for locally advanced rectal cancers tumor (T3-4 N0 or Tany N+) [**9**]. The goals of neoadjuvant radiochemotherapy are to increase local control, pathologic complete response and the rate of sphincter preservation; in addition, it is less toxic than administered postoperative [**9**]. Maybe it would have been better for this patient, whose tumor was cT3 on the MRI performed in August 2011, to receive neoadjuvant radiochemotherapy and according to the postoperative pathological result, adjuvant treatment.

## Conclusion

The tracheobronchial tree should be taken into consideration as a metastatic site for primary solid tumors localized extrapulmonary.

**Source of founding**

This work received financial support through the project entitled “CERO – Career profile: Romanian Researcher”, grant number POSDRU/159/1.5/S/135760, co-financed by the European Social Fund for Sectoral Operational Programme Human Resources Development 2007-2013.

**Disclosures**

Authors declare that there is no conflict of interest regarding the publication of this paper.
